# THE RELEVANCE OF SNAP PROGRAM ACCESSIBILITY FOR FOOD INSECURITY, FOCUSING ON ADULTS WITH DISABILITIES

**DOI:** 10.1093/geroni/igad104.0350

**Published:** 2023-12-21

**Authors:** Laura Samuel, Rhonda Wright, Varshini Varadaraj, Bonnielin Swenor

**Affiliations:** Johns Hopkins University School of Nursing, Baltimore, Maryland, United States; Johns Hopkins University School of Nursing, Baltimore, Maryland, United States; Johns Hopkins University, Baltimore, Maryland, United States; Johns Hopkins University, Baltimore, Maryland, United States

## Abstract

Individuals with disabilities are not only more likely than their peers without disabilities to experience food insecurity, but also encounter disproportionate barriers to Supplemental Nutrition Assistance Program (SNAP) enrollment. This study examined associations between state-level measures of SNAP enrollment accessibility (flexibility, efficiency, and accessibility in the enrollment process; and the presence of either broad-based categorical eligibility or the Combined Application Project) with SNAP participation and food insecurity levels among low-income participants (income ≤185% of the poverty threshold) aged ≥15 years in the 2021 Current Population Survey, both overall (n=19,895) and among those with disabilities (n=3,484). In multi-level models adjusted for individual and state characteristics, living in a state with broad-based categorical eligibility was associated with higher rates of SNAP participation among all low-income individuals (b= 0.300, 95% CI: 0.096, 0.505). Among those with disabilities, having greater flexibility in SNAP enrollment (b= 0.141, 95% CI: 0.007, 0.276) and living in a state with the Combined Application Project (b= 0.271, 95% CI: 0.017, 0.525) were associated with a higher likelihood of SNAP participation. Living in a state with more efficient SNAP enrollment was associated with lower food insecurity levels (b= -0.073, 95% CI: -0.136, -0.010) among all low-income individuals and having broad-based categorical eligibility was associated with lower food insecurity levels (b= -0.237, 95% CI: -0.451, -0.022) among those with disabilities. These findings highlight the importance of SNAP program accessibility to address food insecurity among all low-income Americans and suggest that enrollment barriers may differ for people with and without disabilities.

